# Assessing antimicrobial stewardship governance in Northeast Brazilian hospitals: a survey-based analysis

**DOI:** 10.1093/jacamr/dlae116

**Published:** 2024-08-05

**Authors:** Sylvia Lemos Hinrichsen, Marcela Coelho de Lemos, Juliana Magalhães Bernardino, Juliana Andrade Lima, Genaro Carrazone, Tatiana Vilella, Gabriel Trova, Libia Moura, Reginaldo Gonçalves de Lima-Neto, Adrian John Brink

**Affiliations:** Centro de Ciências Médicas—Departamento Medicina Tropical, Universidade Federal de Pernambuco (UFPE), Pernambuco, Brazil; Instituto Social Medianeiras da Paz (ISMEP), Infectious Disease Department—PSA Nordeste, Pernambuco, Brazil; Instituto Social Medianeiras da Paz (ISMEP), Infectious Disease Department—PSA Nordeste, Pernambuco, Brazil; Instituto Social Medianeiras da Paz (ISMEP), Infectious Disease Department—PSA Nordeste, Pernambuco, Brazil; Instituto Social Medianeiras da Paz (ISMEP), Infectious Disease Department—PSA Nordeste, Pernambuco, Brazil; Instituto Social Medianeiras da Paz (ISMEP), Infectious Disease Department—PSA Nordeste, Pernambuco, Brazil; Instituto Social Medianeiras da Paz (ISMEP), Infectious Disease Department—PSA Nordeste, Pernambuco, Brazil; Laboratório Especial de Microbiologia Clínica (LEMC), Universidade Federal de São Paulo—UNIFESP, São Paulo, Brazil; Centro de Ciências Médicas—Departamento Medicina Tropical, Universidade Federal de Pernambuco (UFPE), Pernambuco, Brazil; Centro de Ciências Médicas—Departamento Medicina Tropical, Universidade Federal de Pernambuco (UFPE), Pernambuco, Brazil; Division of Medical Microbiology, Department of Pathology, Faculty of Health Sciences, University of Cape Town, Cape Town, South Africa; National Health Laboratory Services, Groote Schuur Hospital, Cape Town, South Africa; Faculty of Health Sciences, Institute of Infectious Disease and Molecular Medicine, University of Cape Town, Cape Town, South Africa

## Abstract

**Background:**

Effective governance of antimicrobial stewardship (AMS) and infection prevention control (IPC) in healthcare facilities is crucial for safeguarding patients against healthcare-associated infections and enhancing patient outcomes by optimizing antibiotic use and curbing the spread of antimicrobial-resistant (AMR) pathogens.

**Objectives:**

To assess the current AMS governance in two public hospitals in Northeast of Brazil, specifically focusing on identifying institutional antibiotic policies and operational practices.

**Methods:**

A survey was conducted by team leaders of both hospitals from 2020 to 2022 using a questionnaire adapted from the Pan American Health Organization (PAHO) recommendations for implementing AMS programmes (ASP) in Latin America and the Caribbean, alongside criteria from the National Health Surveillance Agency (ANVISA) and CDC.

**Results:**

Fifty leaders, from senior management to coordinators, answered the questionnaire. Results indicate a lack of AMS process measures, specialist support, systematic antimicrobial utilization analysis and structured IPC programmes, especially in one hospital where patient records remain in paper format.

**Conclusions:**

The empirical use of antimicrobials without local epidemiological or susceptibility data underscores the absence of logistical support for microbiological cultures in the region. These findings emphasize the urgent need for systematic AMS processes and multiprofessional teams to drive AMS and IPC practices, essential for patient care and safety.

## Introduction

The inappropriate use of antibiotics, the increase and spread of antimicrobial-resistant (AMR) pathogens and the lack of new antibiotics represents a major concern for global public health.^1–7^ The emergence of MDR organisms highlights the need for antimicrobial stewardship programmes (ASPs) across all healthcare sectors. The benefit of effective ASPs ensures patient safety and improved clinical outcomes, reduction in costs and mitigating bacterial resistance.^1–7^

While effective ASPs have improved the use of antimicrobials in many high-income countries, such as Australia, England, France, Netherlands, Germany and the USA, implementing ASPs in resource-limited settings and low- and middle-income countries is particularly challenging.^1,2,6,8–11^ Healthcare institutions face significant constraints, including limited infrastructure, high patient load, and a lack of orientation and training on rational antimicrobial management and antimicrobial stewardship (AMS).^1,2,4^

Guidelines for empirical use of antimicrobials should be based on the institution’s epidemiology and are critical for any ASP, but require expertise and training based on international experiences adapted to local realities.^4,10–12^ Protocols for developing and implementing ASPs have been published and they provide recommendations on the prerequisites that should be met when starting an ASP, such as the availability of a multidisciplinary AMS team composed of dedicated and financially compensated core members and the availability of a local antibiotic formulary and antibiotic guidelines.^1,11^

The goal of this study was to identify the existing care practices according to AMS processes and health management policies practised by two hospitals located in Northeastern Brazil. It was also the aim to use the baseline data to leverage support for implementation of AMS by educating and training health professionals, from various health specialties, and to create a multidisciplinary network of leaders capable of promoting, disseminating and monitoring best practices relating to appropriate use of antibiotics.

## Methods

### Study sites

An observational survey was conducted before implementation of ASPs in 2020–2022 in two public hospitals in Northeast Brazil, both located in Chapada do Araripe, Pernambuco, Brazil, with a resident population of approximately 307 000 habitants (rural and urban area) and comprising10 municipalities served by two referral hospitals, including one exclusively for SUS (Brazil’s Unified Health System) patients and the other for SUS and patients with health insurance.

Hospital A is situated in Araripina, Pernambuco, which is approximately 690 km away from the capital of Pernambuco, Recife.^13^ Hospital A has 110 beds, including 10 ICU beds, 10 respiratory ICU beds, 10 paediatric ICU beds and 10 neonatal ICU beds, all of which were established in 2020 during the COVID-19 pandemic. Hospital B is in Ouricuri, in the hinterland of Pernambuco, and occupies an area of 2 382 570 km^2^, which represents 2.25% of the State of Pernambuco.^13^ Hospital B consists of 250 beds in total, with 10 general ICU beds and 10 respiratory ICU beds.

During the period under study, the hospitals did not have standardization of rational use of antimicrobials (e.g. guidelines) and there was also no effective control over the restriction of use for any antimicrobial employed. This was because the Hospital Infection Control Committee was in the process of implementation.

### Ethics

The study has received approval from the Ethics Committee for Research Involving Human Beings of the Federal University of Pernambuco (protocol number 52653421.9.00005208/2021). Additionally, official permission was obtained from all participating hospitals.

### Methods

The aims of this study were to ascertain existing AMS governance in the two public hospitals, before implementation of a formal ASP programme, specifically to identify the existing institutional antibiotic policies and operational practices. The survey was conducted by the principal investigator at both institutions between 2020 and 2022.

#### Questionnaire

A questionnaire (Supplementary Data 1, available as Supplementary data at *JAC-AMR* Online) was adapted according to the Pan American Health Organization (PAHO) recommendations for implementing AMS programmes in Latin America and the Caribbean, the National Health Surveillance Agency (ANVISA) implementation of antimicrobial use management programme and according to the CDC assessment tool for core elements of hospital ASPs.^1,2,7,14,15^ It was validated by the management of both hospitals and presented to team leaders in meetings by the main investigator.

The questionnaire was constructed with the required activities for the use of antimicrobials and infection control, with response options of ‘yes’, ‘no’ and ‘not applicable’. Space was provided for additional comments related to each question. Twelve questions were selected and structured, according to Kallen *et al.*, into three building blocks for effective institutional ASPs: stewardship prerequisites, stewardship objectives and improvement strategies.^7^

Fifty team leaders from both hospitals were invited to respond to the questionnaire, and the responses were compared and taken into consideration to recognize the status of the ASP: whether it needed to be fully implemented or whether it was in progress.

#### Implementation process

The implementation of ASP activities in the hospitals A and B during 2020 and 2022 was based on the institution diagnosis according to baseline survey responses. For that matter, several activities were developed: (i) identification of leaders and multidisciplinary teams and their roles; (ii) diagnostic sheets for assessing existing realities; (iii) recognition of systematized process defined as the organization of elements into coherent groups or according to a specific plan; (iv) creation of new systems and development of clear processes for events and task to minimize misunderstanding and confusion and (v) provision of synchronous and asynchronous training focused on topics addressed in the diagnostic sheets.^7^

## Results

The questionnaire was answered by 100% of those surveyed, made up of 50 team leaders from different areas of both hospitals, such as the infection control service; pharmacy, nursing and medical coordination; the surgical centre; ICU; urgency; laboratory and hospitality, which we consider to be representative of all professionals who work in both hospitals. The survey revealed the absence of AMS governance or implementation of any type of AMS process measures. While there was an infection prevention control (IPC nurse) employed for the hospital’s biosafety procedures and to enable IPC according to the ANVISA (Brazil), no infectious diseases physician, microbiologist and clinical pharmacist support were evident in both hospitals (Table 1).

**Table 1.** Baseline existing AMS governance e Chapada do Araripe during 2020 and 2022

Required activities	Hospitals reality	Comments
Infection disease physician	No	Specialist absent in the region
Infection control practitioner (nurse)	Yes	There is a nurse responsible for the hospital’s biosafety procedures and infection control according to the ANVISA (Brazil)
Microbiologist Available locally Inter-consultation available	No	There is a general support laboratory, but due to the lack of local logistics and the distance from the region to the centres with microbiologic support, culture collection is carried out twice a week with a delivery deadline for results in about 7 to 10 days
Clinical pharmacist General Antimicrobial use specialist	No	There is a pharmacy dispenser
Systematized infection control programme with periodic reports and action plans	No	There are general procedures on infection control according to ANVISA (Brazilian legislation), but with no integrated systematized processes
IHI (Institution of Health Improvement) bundles Ventilator-associated pneumonia Central line-associated bloodstream infections Catheter-associated urinary tract infections	No	There are no systematized processes implemented on infection control related to healthcare assistance
Systematized ASP with daily multidisciplinary bedside visits. If ‘yes’, what frequency: Daily Once a week Once every 2 weeks Once a month Once every 2 months 4 days per year 1 day per year No frequency Other	No	Owing to the lack of ASP, it is not possible to observe whether the use of antimicrobials is in accordance with required and/or recommended standards In the ICUs, the physicians discuss the cases at convenient times without the participation of multidisciplinary teams
Multidisciplinary professionals available in the institution	Yes	Multidisciplinary professionals according to the health institution’s profile Regime of shifts and/or day labourers
Leadership responsible for the use of antimicrobials in nursery. If ‘yes’: In all nurseries In almost all nurseries In 50% of the nurseries	No	In the ICUs, the person responsible for the antimicrobial’ prescriptions is the physician on duty. In the wards, the person in charge is the prescribing physician according to specialties
Type of patient record: Non-existent Paper Electronic Other	Hospital A: paper Hospital B: electronic	
Form for the adequate use of antimicrobial. If ‘yes’: Electronic via Internet Electronic via intranet Electronic via application Printed in a pocket guide Printed in the medical room	No	The antimicrobials are dispensed based on the medical prescription by the various prescribing specialties without the interference or supervision of an ID physician
Use of antimicrobials Empiric Based on microbiology Local protocol Other	Empiric	The health institutions do not have periodic support in microbiology due to the difficulties in the logistics of the collection by local laboratories that outsource this type of exam. There is no antimicrobial use protocol based on institutional guidelines. There is no protocol-based start sequence nor de-escalation The duration of antimicrobial use is not defined, leading to uncontrolled use in many cases

Although there is a general support for the laboratory, due to inefficient logistics and the distance from the region to the centres with microbiologic support culture collection was carried out twice a week with a turn-around time of 7 to 10 days. Therefore, the use of antimicrobials was empirical, unrelated to local epidemiology or susceptibility profiles, due to the lack of antibiograms. While pharmacists were available for dispensing, no antibiotic utilization was assessed nor were any pharmacist-driven AMS interventions evident.

A written systematized IPC programme with periodic reports and action plans was absent in both hospitals, and no IPC bundles had been implemented. Similarly, a systematized ASP programme involving, for example, multidisciplinary bedside engagement was absent. Notably, multidisciplinary professionals were available in the institutions but were not involved in ASP activities and no neonatal leadership was responsible for the use of antimicrobials in nurseries.

## Discussion

To our knowledge, this is the first observational investigation into the existence of AMS governance in Northeastern Brazil, specifically in the Chapada do Araripe region of Pernambuco. This research is significant as it sheds light on the reality of AMS implementation in this region and the hospitals under investigation, providing valuable insights for the future development and implementation of healthcare assistance processes, particularly concerning antibiotic use.

Studies have highlighted challenges encountered when implementing ASP, including a lack of interdepartmental coordination, communication and awareness, contributing to antimicrobial resistance.^15–20^ Many barriers hinder the implementation of ASPs in public and private hospitals in Brazil, especially in the Northeastern region. It was observed that in the studied hospitals there was a designated nurse responsible for procedures associated with infection control, however, challenges such as limited culture collection frequency and extended delivery time for results pose a significant obstacle to treatment and infection control.

The absence of integrated processes for infection control and ASP indicates a gap in the healthcare system’s ability to manage effectively infectious diseases and antimicrobial use. Lack of ASP oversight and suboptimal antimicrobial use without infectious disease physician involvement contribute to antimicrobial resistance.^19,20^

The discussion of cases in ICU by physicians without the multidisciplinary team support and institutional guidelines for antimicrobial use underscores the need for structured approaches to managing antimicrobial therapy and infectious disease. Hospital managers play a crucial role in promoting safety, but in public hospitals, especially in rural areas, they tend to be more reactive than proactive, focusing on compliance and infrastructure development.^16,17,19,20^

## Limitations

The study was conducted in a limited number of rural hospitals, which may not fully represent the diverse healthcare landscape in the Northeast region of Brazil.

Furthermore, this survey focused on assessing the in-hospital systematic processes related to antibiotic use and did not differentiate between specific multidisciplinary teams of patient groups. Thus, its scope may not fully encompass all relevant aspects of IPC and ASPs. Future research endeavours should aim to expand the survey to cover other important determinants to gain a comprehensive understanding of these areas.

### Conclusions

It is evident that there is a pressing need to prioritize the implementation of AMS in both hospitals. It is imperative to equip healthcare professionals across various specialties with adequate education and training to ensure a comprehensive understanding of AMS principles, in addition to understanding local processes and limitations. Additionally, establishing a multidisciplinary network of leaders who can promote, disseminate and monitor best practices regarding the judicious use of antibiotics is essential.

As healthcare professionals fulfil distinct roles in patient care, it is essential to delineate their responsibilities within the framework of ASPs and the prevention or healthcare-associated infections. In this context, the inclusion of an infectious diseases’ physician, microbiologists and clinical pharmacists in our study hospitals emerge as crucial for effective change management. These professionals are poised to drive the implementation of an ASP and foster multidisciplinary collaboration, thereby enhancing the efficacy of AMS initiatives.

## Supplementary Material

dlae116_Supplementary_Data

## Supplementary data

Supplementary Data 1 available as Supplementary data at *JAC-AMR* Online.
