# Supplementary material for: Assessing antimicrobial stewardship governance in Northeast Brazilian hospitals: a survey-based analysis
Source: JAC Antimicrob Resist. 2024 Aug 5;6(4):dlae116. doi: 10.1093/jacamr/dlae116 (PMC11299941; doi:10.1093/jacamr/dlae116)
Supplement: dlae116_Supplementary_Data [file dlae116_supplementary_data.docx]

**Supplementary data**

**Supplementary data 1.** Questionnaire: required activities for the use of antimicrobials in infection control

| Required activities | Hospitals reality (yes/ no/ not applicable) | Comments |
| --- | --- | --- |
| Infection disease physician |  |  |
| Infection control practitioner (nurse) |  |  |
| Microbiologist   - Available locally - Inter-consultation available |  |  |
| Clinical pharmacist   - General - Antimicrobial use specialist |  |  |
| Systematized infection control program with periodic reports and action plans |  |  |
| IHI (Institution of Health Improvement) bundles   - Ventilator-associated pneumonia - Central line-associated bloodstream infections - Catheter-associated urinary tract infections |  |  |
| Systematized Antimicrobial Stewardship Program (ASP) with daily multidisciplinary bedside visits. If “yes”, what frequency:   - Daily - Once a week - Once every two weeks - Once a month - Once every two months - Four days per year - One day per year - No frequency - Other |  |  |
| Multidisciplinary professionals available in the institution |  |  |
| Leadership responsible for the use of antimicrobials in nursery. If “yes”:   - In all nurseries - In almost all nurseries - In 50% of the nurseries |  |  |
| Type of patient record:   - Nonexistent - Paper - Electronic - Other |  |  |
| Form for the adequate use of antimicrobial. If “yes”:   - Electronic via internet - Electronic via intranet - Electronic via application - Printed in pocket guide - Printed in the medical room |  |  |
| Use of antimicrobials   - Empiric - Based on microbiology - Local protocol - Other |  |  |
